# Recurrence and Coniglobus Volumetric Resolution of Subacute and Chronic Subdural Hematoma Post-Middle Meningeal Artery Embolization

**DOI:** 10.3390/diagnostics11020257

**Published:** 2021-02-07

**Authors:** Ambooj Tiwari, Adam A. Dmytriw, Ryan Bo, Nathan Farkas, Phillip Ye, David S. Gordon, Karthikeyan M. Arcot, David Turkel-Parrella, Jeffrey Farkas

**Affiliations:** 1 Interventional Neuro Associates, 43 Westminster Avenue, Bergenfield, NJ 06721, USA; ryanbo@gmail.com (R.B.); Nathan.farkas@gmail.com (N.F.); phillip.ye1@gmail.com (P.Y.); karcot@intneuro.org (K.M.A.); dtparrella@intneuro.org (D.T.-P.); farkas@intneuro.org (J.F.); 2 Neuroradiology & Neurointervention Service, Brigham and Women’s Hospital, Harvard Medical School, Boston, MA 02215, USA; 3 Departments of Neurology, Radiology & Neurosurgery, NYU School of Medicine & New York University Langone Health, Brooklyn, NY 11220, USA; david.gordon2@nyumc.org; 4 Department of Vascular Neurology & Neurointerventional Surgery, NYU Grossman School of Medicine, 150 55th Street, Brooklyn, NY 11220, USA; 5 Department of Neurology, Washington University at St. Louis, 660 S Euclid Ave, St. Louis, MO 63110, USA

**Keywords:** volumetric imaging, subdural hematoma, embolization, cerebral angiography, neuroradiology

## Abstract

Objective: To study the efficacy of middle meningeal artery (MMA) embolization for the treatment of chronic subdural hematoma (SDH) and characterize its post-embolization volumetric resolution. Methods: Ten patients diagnosed with 13 cSDH underwent MMA embolization. SDH volumes were measured from time of initial discovery on imaging to pre-operative, post-operative, short-term and long-term follow-up. Time between procedure to obliteration was also measured. Volumetric analysis was done using the coniglobus formula, and recurrence rate as well as resolution timeline was defined using best-fit models. Results: Out of 10 patients, five were recurrent lesions, three were bilateral and seven unilateral cSDH. Average and median pre-operative volumes were 105.3 cc and 97.4 cc, respectively. Embolization on average was performed 21 days after discovery. Sixty percent of patients had concurrent antiplatelets or anticoagulation use. Forty percent underwent embolization treatment as the primary therapy. Recurrence was not seen in any patients treated with embolization. There were no peri- or post-operative complications. Five patients experienced complete or near-complete obliteration, while those with partial resolution showed a composite average of 75% volumetric reduction in 45 days. Post-embolization, the volumetric resolution followed an exponential decay curve over time and was independent of initial volume. Conclusion: MMA embolization contributed to a marked reduction in SDH volume post-operatively and can be used as a curative therapy for primary or recurrent chronic SDH.

## 1. Introduction

In spite of effective treatment of subdural hematoma, recurrence is seen in approximately 10–20% cases over an average interval of 1–8 weeks [1,2,3,4,5]. This is primarily due to the pathophysiology of the neo-membrane formed within the hematoma. Although burr hole drainage resolves the volumetric pressure of the hematoma, it does not address the underlying pathophysiology of inflammation, neovascularization and re-hemorrhage [6,7]. This may particularly be at play in cases associated with higher rates of recurrence: patients with cerebral atrophy, congenital or acquired coagulation disorders (chronic renal failure), use of antiplatelet or anticoagulant drugs and delayed time to treatment [7,8,9].

The development of chronic subdural hematoma (cSDH) is hypothesized to result from inflammation and the promotion of angiogenic factors secondary to prolonged dural insult [10]. Micro-hemorrhage from fragile neovascular channels formed within the hematoma membrane is the putative contributor to its collection or recurrence. [2,11] This angiogenesis is often associated with the middle meningeal artery (MMA), whose consequent hypertrophy is often used as a surrogate for this pro-angiogenic response [12].

Neurological injury in cSDH is typically reversible and often associated with direct compression from the subdural hematoma. The latter appears to be predominantly associated with direct compression/tension of the subdural hematoma [10]. Other putative mechanisms revolve around specific regional decrease in blood flow to the thalamus and basal ganglia regions. The consequent impaired thalamic function is then implicated in a spreading depression that further impairs various cortical regions that can present as focal or global neurological deficits [8]. Another proposed mechanism for dysfunction of neurological tissue appears to be related to local perfusion derangements in the tissue adjoining the hematoma and possibly further complicated by venous congestion [11]. Finally, direct intracranial pressure (ICP) derangement is typically seen in a delayed fashion secondary to the slow development of hematoma. This is because most such cases are associated with cerebral atrophy which compensates for the volume enlargement until late in the disease, and even in the presence of a midline shift. However, when these compensatory mechanisms are overwhelmed, the volumetric compliance of the intracranial cavity is compromised and even small increases in intracranial volume are associated with larger increases in ICP. This then created a vicious cycle of decreased cerebral perfusion and global cerebral ischemia. It is also possible that the latter may play a significant role in acute on chronic SDH [12].

Several operators have successfully employed poly-vinyl alcohol (PVA) or n-butyl-2-cyanoacrylate (NBCA) particles for embolization of the neovascular channels associated with MMA. [2,8,11,13,14] This targets the underlying mechanism and has been successfully attempted both as primary and adjuvant therapy for recurrent cSDH. The procedure is deemed to be safe, and post-embolization recurrence has a low incidence. It has been particularly useful in patients who are not good surgical candidates or are poor responders to traditional surgery [4,14,15].

However, a clear characterization of the volumetric and temporal resolution has never been attempted. We present our experience of both the safety and efficacy of the procedure, especially given the unique use of tris-acryl gelatin microsphere (Embosphere, MERIT MEDICAL, South Jordan, UT, USA) for our embolization procedures. We also report the post-embolization rate of recurrence as well as try to define a timeline for volumetric improvement.

## 2. Materials and Methods

### 2.1. Patient Selection

We performed a retrospective review of our neuro-interventional database. Patients undergoing MMA embolization for subdural hematoma were included for the purposes of this paper. Patients with following subdural characteristics were included: subacute or chronic, new or recurrent, post burr-hole surgery or primary, unilateral or bilateral. Exclusion criteria were those with an acute subdural hematoma as well as asymptomatic patients. This yielded 10 patients with 13 subdural hematomas who underwent MMA embolization.

### 2.2. Data Collection

Variables were collected among four categories: clinical, radiological, angiographic/procedural and outcome. Clinical variables were as follows: demographics, clinical presentation, time of discovery, new or recurrent, primary or post-evacuation, use of anticoagulation or antiplatelet medications, presence of any other coagulopathies and type of index event causing subdural hematoma.

Radiological data included cSDH volume on pre-op, immediate post-op (0–7 days), peri-op (8–30 days post procedure), short-term (31–90 days) and long-term (91+ days) follow-up non-contrast CT (NCCT) of the head, respectively. For all the patients, volumetric analysis was done based on the coniglobus formula, as has been used in other studies, = ½ × the longest diameter of the hematoma layer with the largest area on axial cuts (cm) × the diameter perpendicular to the longest diameter mentioned (cm) × the thickness of the hematoma (cm) [16]. We also collected information on laterality, density, mass effect on cortical tissue (defined as presence of midline shift), chronicity of hematoma (subacute vs. chronic) and presence of neo-membrane. We also used qualitative descriptions of cortical atrophy which were categorized based on the Pasquier scale [15] (Table 1).

Angiographic/procedural variables included capillary bed hypervascularity, MMA diameter and use of gel foam pledget/coils, in addition to 300–500 micron particles Embospheres. In cases with unilateral cSDH, ipsilateral MMA was compared to the MMA from the unaffected side. In cases with bilateral cSDH, both MMAs were reported independently.

The primary outcome was recurrence on NCCT. The secondary outcome measures include complete vs. partial resolution, temporal analysis of volumetric reduction in cases of partial resolution, as well as procedural complications. We defined resolution as complete or near complete when hematoma volume was less than 10 cc. Partial resolution was defined as SDH volume greater than 10 cc.

### 2.3. Embolization Therapy

For the endovascular embolization procedure, all patients were placed under generalized endotracheal anesthesia. A 5/6 French sheath was used to obtain access to the right common femoral or right radial artery. Thereafter, a 5/6 French diagnostic catheter was used to obtain pre-embolization angiographic imaging to document the presence of hypervascularity as well as MMA hypertrophy. This was done bilaterally in all cases whether the lesion was identified as unilateral or bilateral. The diagnostic catheter was either used as the guiding catheter or exchanged for an intermediate catheter for embolization. This catheter was then advanced into the external carotid artery on the side of the lesion. A microcatheter was used to obtain access to the middle meningeal artery. Diagnostic information was obtained both from the guide catheter in proximal external carotid artery (ECA) and microcatheter in the proximal MMA, especially to identify dangerous internal carotid artery (ICA) branch anastomoses. Typically, a 14 or 21 neuro-endovascular coiling microcatheter was used for the embolization procedure. Microcatheter imaging was also used to identify various branches of the MMA as well as target branches for embolization. The microcatheter was used to selectively catheterize the MMA branch identified for target embolization. Systemic heparinization was performed to keep the activated clotting time (ACT) above 200U. A ratio of 80–90% contrast and 10–20% saline was used to dilute 300–500 µm Embospheres. We performed continuous agitation of the syringe to keep the Embospheres suspended in the saline/contrast mix. Progressive embolization of the branches of the MMA was performed until hypervascularity disappeared and there was slow contrast filling in the main stem of the branch being embolized.

This procedure was repeated for each of the branches supplying the area of the hyper-blush. In some cases, the embolization was concluded either by pushing a gel-foam pledget in the stem or by coiling. Post embolization, the microcatheter was removed under negative suction. The guide catheter was removed, and follow-up angiography was performed with a fresh diagnostic catheter. Diagnostic imaging was performed for both extracranial and intracranial circulations. Post procedure, general anesthesia was reversed, and the patient transferred to the post-operative unit. Routine neuro and neurovascular checks were performed to look for any post-operative neurologic or peripheral complications. A post-operative CT scan was also performed in the next 24–48 h to look for any silent ischemic insults or changes in the appearance of the subdural hematoma.

### 2.4. Statistical Analysis

Demographic and clinical data were analyzed using descriptive statistics including frequency, percentage, median value and standard deviation. Several radiological data points including complete radiological resolution (on CT scans) or average composite partial resolution and its timeline were also reported using descriptive statistics. Recurrence between historical controls and MMA embolization were compared using the Wilson Score Interval, and the recurrence rate between both methods presented within 95% and 99% confidence intervals. These historical controls were obtained from studies on the natural history and nonsurgical management of subdural hematomas [16,17,18,19]. Finally, individual graphs of volumetric reduction in hematoma were graphed against time for each patient. Thereafter a single best-fit model was chosen using the 12-fold cross validation method to find a single model that would best describe the volumetric resolution over time. Two important assumptions were made to arrive at this model. The first that all patients recover in a similar fashion; i.e., the mechanism of the volume reduction for each patient is similar. The second that volumetric reduction in each case follows an “exponential decay” pattern, since a-priori observation from each individual volume–time graph had followed a similar pattern.

## 3. Results

Baseline characteristics of demographic and clinical variables for patients included in the study are summarized in Table 1. The average and median age were 71.4 and 68, respectively. The average and median pre-operative volumes were 105.3 cc and 97.4 cc, respectively. A total of 60% of patients had concurrent use of antiplatelets or anticoagulation at the time of initial insult. A total of 60% underwent embolization for recurrent SDH, while 40% received it as a primary treatment. All patients were symptomatic, of which chronic headaches (70%) were the most common. A total of 80% of cases had midline shift, while more than half (54%) had cortical surface compression. Bilateral embolization was performed for three patients.

Recurrence was not seen in any of the patients treated with embolization. There were no peri or postoperative complications. Five patients reported complete or near complete obliteration of their SDH. Those with partial resolution showed a composite average of 75% reduction in volume at 45 days following the procedure. Case 1 and 8 were both co-incidentally given subcutaneous heparin in the immediate perioperative period for deep venous thrombosis prophylaxis by the non-neurological team members, but subsequently discontinued in the postoperative period by the neuro-endovascular team. The radiological and procedural details are summarized in Table 2.

We utilized the Wilson Score Interval to estimate the recurrence rate of the post-embolization patients compared to historical controls. For embolization, the Wilson Score Interval yielded a 95% confidence interval of the recurrence rate between 0 and 0.125 (or 0–12.5%) and a 99% confidence interval of 0–0.197 (or 0–19.73%). The traditional method, on the other hand, had a 95% confidence interval between 0.118 and 0.160 (or 11.8–16.0%) and 99% confidence interval of 0.113–0.168 (or 11.3–16.8%).

**Figure 1:**
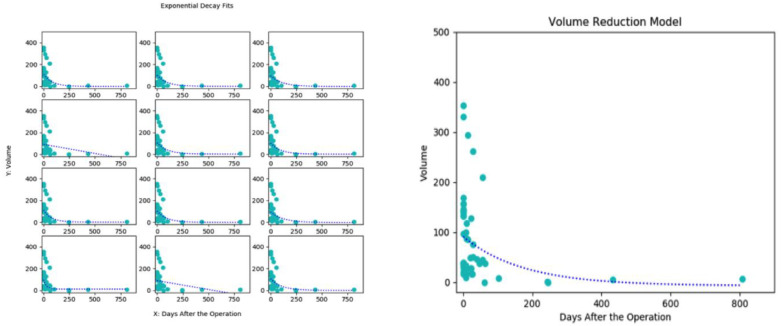
All twelve coniglobus models were first plotted (**left**). After cross-validation, the best fit based on the sum of the residual squares was synthesized depict volume decay (**right**).

We used a 12-fold cross validation to find the best fitted model of volumetric resolution over time. A matrix of the individual graph for each patient is shown in Figure 1 (left panel). A zoomed-in graph of the best model (graph 9 above) is shown in Figure 1 (right panel). We found that the graph followed an exponential decay curve whose formula was dependent on time and was as follows: volume = 98.1 e ^(−0.00576 × Days)^ − 6.74. Thus, the volume decay was independent of initial volume and followed the same pattern over time.

**Figure 2:**
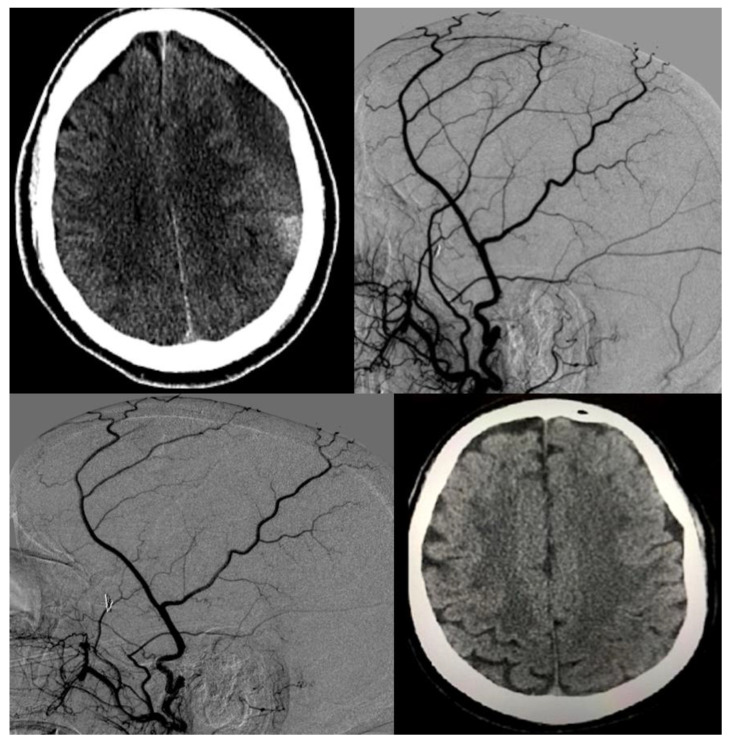
A 68-year-old man with medical history of hypertension and chronic non-valvular atrial fibrillation on coumadin had a minor motor vehicle accident with whiplash injury but no loss of consciousness. Post-trauma emergent non-contrast CT (NCCT) of the head revealed no evidence of subdural hemorrhage. Patient was asked to resume his normal activities as well as continue with anticoagulation regimen. Three weeks later while vacationing in another state, the patient developed subtle speech difficulties, gait disturbance as well as occasional memory lapses. Emergent NCCT was done which revealed bifrontal subdural hematoma (left worse than right) with acute on chronic component and midline shift. He underwent bifrontal burr hole surgeries but had a fresh re-hemorrhage a week later. The patient flew back to home state where another NCCT was prompted by clinical worsening, including coordination difficulties and word-finding difficulties. This revealed significant worsening from acute on chronic SDH as well as midline shift (top left). Patient was taken for emergent MMA embolization, and the images shows pre-embolization (top right) and post-embolization (bottom left) angiograms. Patient then had monthly clinical and radiological (NCCT) follow-ups. Anticoagulation was stopped throughout this period. Four months later, the patient had near resolution of his SDH after which antiplatelets were started. After another month, anticoagulation was started. The last control NCCT was done 3 months after resumption of anticoagulation and is shown in the image on the bottom right.

The pathophysiology of the initial volume depends on patient characteristics, as has been described in natural history studies on subdural hematoma. Therefore, patients with larger volumes had a lower chance of complete resolution as shown by our composite average numbers. A case illustration of post-embolization recovery is shown in Figure 2.

## 4. Discussion

Chronic SDH management can be highly varied, from conservative management with corticosteroids to invasive neurosurgical intervention [20,21]. Current literature supports the use of burr hole trephination and percutaneous bedside drainage as gold standards due to reduction of complications compared to craniotomy, although craniotomy has shown superiority in the prevention of recurrence (Supplementary Tables S1–S3 [1]. Recurrence after burr hole trephination is approximately 20%, with many patients having to undergo multiple repeat procedures before definitive cure [22]. This high rate of recurrence may be a result of inflammatory mediators and fibrinolytic factors within the hematoma space that may be left behind, especially in burr hole drainage without irrigation. [6] Additionally, recurrent surgeries are associated with a higher risk of surgical site infections which in turn can lead to further repeat surgical procedures and consequently worsening functional outcomes [12].

Most recent studies support the initial development of cSDH as initially caused by dural injury promoting chronic inflammation. In cases of traumatic etiology, this rupture of bridging veins between periosteum and dura is what causes the initial collection of blood within the subdural space [8,10]. This mixture of blood and cerebrospinal fluid (CSF) within the cavity, along with prolonged inflammatory response, stimulates dural border cell proliferation, granulation tissue formation and macrophage deposition, all which contribute to the formation of the hematoma membrane [8,10,14]. Studies that focus on the histopathology of a mature subdural membrane reveal 2–3 distinct layers. The inner layer primarily consists of small neo-vascular channels and inflammatory cells such as macrophages and polymorpholeukocytes (PMLs). This connects to the outermost layer through sinusoids. The outermost layer has been observed to consist of macrophages and giant capillaries, which connect to the terminal ends of the MMA. A leak from the sinusoids and rupture of this vascular network contributes to the recurrence of the SDH, which in turns begins to cause MMA hypertrophy, as reported in many studies [23,24]. Super-selective angiography in some cSDH cases has reported cotton wool-like staining at the peripheral end of the MMA, which is speculated to be communicating vessels of this outer membrane and makes the artery a prime target for embolization to prevent subdural collection [25]. In addition, several other markers such as aquaporin-1, a water channel, are also heavily upregulated in the outer membranes and contribute to fluid accumulation and volumetric expansion of CSDH growth [22].

MMA hypertrophy and cotton wool spots (neovascularization) contribute to hyperpermeability, and consequently to persistent bleeding and/or transudate formation. Therefore, MMA embolization may not only be a target for prevention of recurrence, but also a potential for primary therapy for hematoma resorption [2,14]. Most early studies explored the use of MMA embolization for treatment of recurrent cSDH as an adjunct to conventional therapy. The use of embolization was found to have high success rates in the reduction of hematoma volume and significance in prevention of recurrence following conventional therapy [2,8,15]. When compared to conventional therapy, no significant difference was found in functional outcomes for the treatment of recurrent SDH. In addition, embolization therapy had no peri-operative complications when compared to conventional therapy [14]. Half of our cases included patients who underwent MMA embolization for recurrent SDH either as a stand-alone therapy (4 patients, 6 hematomas) or as an adjunct to conventional therapy (1 patient, 1 subdural). Like previous embolization studies, we also had no peri- or post-operative complications in our study.

Few reports show recurrence of SDH after embolization. One case report presented a possible etiology of recurrence 3 months after embolization with craniotomy delaying the healing of the SDH, allowing for additional development of collateral flow from the residual hematoma. Another study showed treatment failure in a similar patient in which recollection occurred post-motor vehicle accident (MVA) requiring craniotomy and evacuation [2,15]. The recurrence rate in our study was 0% and was consistent with other embolization studies and superior to conventional therapy where recurrence has been reported up to 20%. When we combined our radiological recurrence outcomes with data from other embolization studies, we found that recurrence rates for the MMA embolization method were lower when compared to traditional therapies within both 95% and 99.5% confidence intervals [7,8,13,19]. The traditional methods included a composite of both surgical and medical therapies [5,14,17,20,21,22,26,27,28].

Ban et al. were the first to report the use of this technique as a primary therapy for treatment of recurrent cSDH. They showed spontaneous and complete resolution in all asymptomatic patients who underwent MMA embolization as sole treatment, as well as a 2.2% recurrence rate for symptomatic patients who underwent embolization with direct hematoma removal [2]. Link et al. was the first to use this technique for primary treatment of index SDH as well as to report volumetric data [19]. In our data, the other half of our patients (8 hematomas in 5 patients) had MMA embolization as primary treatment for the index cSDH. When compared to historical controls, we also found that rate of recurrence in our study was similar to other embolization studies and superior to other forms of therapy (non-surgical, surgical and observational). [5,22,27]

Complete resolution or near complete resolution was seen in 5 out of 10 patients. When compared to medical management and natural history, our rate was 50% compared to 27.5% and 26%, respectively [14,16,17,26,29]. Xu et al. in their surgical series reported a complete resolution in 55% of their cases and were comparable to our rates. [18] Our rates were similar to other embolization studies that reported rates between 33% and 55%. [8,13,19,30,31]. The average follow-up in our study was 5.7 months or 174 days and was consistent with the other embolization open surgical studies and non-surgical studies whose follow up periods ranged from 2–8 months, 3 months and 1.5–6 months, respectively.

The volumetric resolution model in our case series followed an exponential decay that was independent of volume and only depended on time. Thus, larger volumes tend to have a lower likelihood of complete resolution. This may have been why our complete resolution was only 50% since our average initial volumes tended be larger at baseline compared to historical studies, including both non-surgical and embolization studies [16,18,32,33]. This could have also been the reason why we had a higher incidence of cortical compression (54%) and/or mass effect (80%), as well as a relatively high number of symptomatic patients.

The exponential decay was useful in other ways since it led to a significant reduction in volume in the immediate post-operative period (0–30 days). Our composite volumetric reduction was 75% over an average of 45 days, while in 40% of our cases the resolution continued beyond 90 days. One of the advantages of having a faster resolution timeline is the consequent faster resolution of cortical compression and/or mass effect. Embolization has been associated with shorter brain re-expansion time when compared to patients who were managed through conventional treatment [34,35]. This has also been an important factor in the reduction of recurrence [14]. This is of particular relevance in patients with comorbidities, which do not allow for a conventional approach. Thus, if post-embolization volumetric reduction can mimic hematoma evacuation it may become a viable, less invasive and safer first therapy for non-acute SDH. To our knowledge, this is the first study that has quantified hematoma volumetric resolution as an independent outcome unrelated to recurrence (Supplementary Table S4).

The agent most commonly used for embolization has been 150–250 µm polyvinyl alcohol particles [14,19,36]. Some operators have also used 15–20% n-butyl-2-cyanoacrylate (NBCA) particles [8]. The use of these embolization agents has been in conjunction with gel foam or coil use to occlude the flow in the parent vessel [7,15]. To our knowledge this is the first use of tris-acryl gelatin microspheres, also known as Embosphere (MERIT MEDICAL, South Jordan, UT, USA), for this purpose. The use of coils or gel foam was about 90% in our data as a final embolization step.

The major limitation of this study is the size of the patient population, and future studies would ultimately benefit from large-scale randomized controlled trials. Although some comparison has been made to more traditional surgical interventions for cSDH management, the efficacy of MMA embolization must also be further studied in comparison to conservatively managed patients with spontaneous resolution [2,14]. Given the sparsity of data and the assumptions of the model, it is important to be cautious of the generalizability of our model. Nevertheless, we feel it shines some light on the recovery mechanism of patients after embolization. Although the preponderance of current literature has shown minimal to no recurrence of cSDH post embolization, factors that may contribute to recurrent cases also require study [15]. Of note is that we were not able to compare long term outcomes as we had limited follow-up in our study beyond 6 months. We also cannot exclude the possibility of selection bias since we may have had a higher selection of symptomatic patients and more recalcitrant cases which would have had a higher likelihood of being considered for therapy due to persistence of their symptoms.

## 5. Conclusions

Endovascular embolization of the middle meningeal artery can be used as an effective means to achieve volumetric reduction and resolution of subacute or chronic primary or recurrent subdural hematoma with low rates of recurrence post-embolization. Large scale trials with attention to functional outcomes are needed.

## Figures and Tables

**Table 1:** Demographic and Clinical Details.

Case No.	Chronic Alcoholism	Sulcal Effacement	Mass Effect	Age	Global Cortical Atrophy Scale *	Hepatic Dysfunction	Anticoagulant/ Anti-Platelets			History of Carcinoma	Dialysis	Coagulopathy
							Pre-Op	Peri-Op	Post-Op			
1	N	Y	Y	45	0	N	N	Y	N	N	N	N
2	N	Y	Y	68	1	N	Y	N	Y	N	N	N
3	N	N	N	63	1	N	Y	N	N	N	N	N
4	N	N	N	63	1	N	Y	N	N	N	N	N
5	N	Y	Y	96	3	N	N	N	N	N	N	N
6	N	Y	Y	96	3	N	N	N	N	N	N	N
7	N	Y	Y	86	2	N	Y	Y	N	Y	N	Y
8	N	Y	N	52	0	N	N	Y	N	N	N	N
9	N	N	N	93	2	N	N	N	N	N	N	Y
10	N	N	N	93	2	N	N	N	N	N	N	Y
11	N	Y	Y	68	0	N	Y	N/A	Y	N	N	N
12	N	N	Y	75	2	N	N	N	N	N	N	N
13	N	N	Y	68	0	N	N	N	N	N	N	Y

* Pasquier Scale; 0: normal volume/no ventricular enlargement; 1: opening of sulci/mild ventricular enlargement; 2: volume loss of gyri/moderate ventricular enlargement; 3: “knife blade” atrophy/severe ventricular enlargement.

**Table 2:** Procedural and Radiological Timelines.

Case No.	Discovery to Embolization (Days)	Previous Surgery	Pre-Op	Post-Op (1–6 Days)		Peri-Op (7–30 Days)				Short Term (30–90 Days)		Long Term (90 Days+)	
			Volume (cc)	Interval	Volume (cc)	Interval	Volume (cc)	Interval	Volume (cc)	Interval	Volume (cc)	Interval	Volume (cc)
1	37	Y	29.4	2	28.8	25	16.25					433	5.6
2	53	Y	97.44	2	97.5					46	37.265	243	1.08
3	7	N	34.11	3	35.43	7	28.61						
4	7	N	33.31	3	34.4	7	33.41						
5	4	N	144	1	144	14	85.5	28	76	56	44.8		
6	4	N	353.43	1	331	14	294	28	262	56	210		
7	15	N	133.92	1	132.48	10	118.8			39	46.8	102	8.64
8	24	N	21.26			9	18.9	27	16.45			808	6.8
9	6	Y	38.55	1	39.2	23	28						
10	6	Y	158.18	1	146.3	23	127.5						
11	23	N	168.75	0	156.56	7	99.36	20	49.8				
12	72	Y	140.3	1	137.51	9	87.5	28	50.25	63	37.56		
13	12	Y	17			8	9.3			62	0	245	0

## Data Availability

Data are available via online supplements and upon reasonable request.

## Supplementary Materials

The following are available online at https://www.mdpi.com/2075-4418/11/2/257/s1, Table S1: Recurrence Rates (Historical Controls); Table S2: Volumetric Resolution (Historical Controls); Table S3: Patient Demographics and Clinical Characteristics; Table S4: Wilcoxon Score Index based Recurrence Rates.
