# Supplementary material for: Recurrence and Coniglobus Volumetric Resolution of Subacute and Chronic Subdural Hematoma Post-Middle Meningeal Artery Embolization
Source: Diagnostics (Basel). 2021 Feb 7;11(2):257. doi: 10.3390/diagnostics11020257 (PMC7915255; doi:10.3390/diagnostics11020257)
Supplement: Supplementary file 1 [file diagnostics-11-00257-s001.pdf]

Supplementary Table S1: Recurrence Rates (Historical Controls)			
	No. of Cases	Recurrence	Recurrence Percentage
**Embo Only**			
Hashimoto et al	4	0	0.00%
Hirai et al	2	0	0.00%
Link et al	6	0	0.00%
Mino et al	2	0	0.00%
Tiwari et al	13	0	0.00%
Total	27	0	0.00%
**Burr Hole Drainage with and without Irrigation**			
Mori et al	499	48	9.62%
Torihashi et al	337	61	18.10%
Santarius et al	215	36	16.74%
Total	1051	145	13.80%
**Craniotomy**			
Lee et al	134	25	18.66%
Robinson et al	10	2	20.00%
Total	144	27	18.75%
**Medical Management**			
Delgado-Lopez et al	101	28	27.72%
Pichert et al	56	8	14.29%
Poulsen et al	47	0	0.00%
Total	204	36	17.65%
**Observation Only**			
Kim et al	16	3	18.75%
Sun et al	4	2	50.00%
Parlato et al	5	0	0.00%

Lusins et al	9	1	11.11%
Total	34	6	17.65%

**Supplementary Table S2: Volumetric Resolution (Historical Controls)**						
**Embolization**	**Number of Cases**	**Complete Reduction**	**Partial Reduction**	**No Change**	**Increase in Volume**	**Average Follow-Up Period (months)**
Hashimoto et al	2	1	1	0	0	3.50
Hirai et al	2	1	0	1	0	8.00
Link et al	6	2	4	0	0	2.33
Mino et al	2	0	2	0	0	6.00
Tiwari et al	13	4	8	1	0	5.70
**Total**	25	8	15	2	0	4.92
**Surgical**						
Xu et al	63	35	16	12	0	3.00
**Total**	63	35	16	12	0	3.00
**Medical Management**						
Wang et al	23	17	5	0	1	3.00
Poulsen et al	47	0	47	0	0	1.50
Kageyama et al	21	8	12	0	1	1.93
**Total**	91	25	64	0	2	1.98
**Observation**						
Kim et al	16	13	0	0	3	4.25
Parlato et al	24	5	NR	NR	NR	1.50
Lusins et al	9	0	9	0	0	1.50
Lee et al	40	2	NR	NR	NR	1.50

Naganuma	4	4	NR	NR	NR	6.74
**Total**	93	24	9	0	3	2.20

**Supplementary Table S3: Patient Demographics and Clinical Characteristics**	
	N = 10
Average Age	71.40 ± 16.58
Gender (M:F)	7:3
Trauma	5
Antiplatelet or Anticoagulant Use	6
Prior Surgical Hematoma Removal	5
Symptomatic	10
Bilateral Hematomas	3
Unilateral Hematomas	7

**Supplementary Table S4: Wilcoxon Score Index based Recurrence Rates**		
	**95% Confidence**	**99% Confidence**
**Embolization**	0%-12.5%	0%-19.73%
**Traditional Method**	11.8%-16.0%	11.3%-16.8%
